# Case Report: Early diagnosis of lethal multiple pterygium syndrome with micrognathia: Two novel mutations in the *CHRND* gene

**DOI:** 10.3389/fgene.2023.1005624

**Published:** 2023-01-17

**Authors:** Caiyuan Chen, Jin Han, Jiaxin Xue, Ru Li, Guilan Chen, Xin Yang, Jiajie Tang, Fucheng Li, Dongzhi Li

**Affiliations:** ^1^ Prenatal Diagnosis Center, Guangzhou Women and Children’s Medical Center, Guangzhou Medical University, Guangzhou, China; ^2^ School of Information Management, Wuhan University, Wuhan, China

**Keywords:** lethal multiple pterygium syndrome, CHRND gene, micrognathia, prenatal diagnosis, first trimester

## Abstract

Lethal multiple pterygium syndrome (LMPS) is a rare disease with genetic and phenotypic heterogeneity and is inherited in an autosomal recessive (AR) pattern. Here, we have presented clinically significant results describing two novel mutations of *CHRND* gene: NM_000751.2: c.1006C>T p.(Arg336Ter) and NM_000751.2:c.973_975delGTG p.(Val325del), and measurement of the facial angle for determining micrognathia by prenatal diagnosis in the first trimester of pregnancy for a Lethal multiple pterygium syndrome case. In conclusion, this report complements the spectrum of genetic variants and phenotype of Lethal multiple pterygium syndrome and provides reliable recommendation for the counseling of future pregnancies in families with the disease.

## Introduction

Lethal multiple pterygium syndrome (MIM 253290), a rare disorder, was first reported by Gillen and Pryse-Davis. And it is one of the types of multiple pterygium syndrome (MPS) (Brink et al., 2003; Morgan et al., 2006), with an incidence of <1/100000, which has not been assessed concretely (Orpha, 2019). Either autosomal or X-linked recessive inheritance has been suggested, and the latter is rarely reported (Tolmie et al., 1987; Hertzberg et al., 2000). LMPS is distinguished by multiple pterygium, arthrogryposis and akinesia, nuchal cystic hygroma, hydrops fetalis, intrauterine growth retardation, and death (de Die-Smulders et al., 1990b). Furthermore, other detailed manifestations such as micrognathia, low-set ears, and cleft lip and palate have also been noted (Meizner et al., 1993). Three clinical subtypes of LMPS have been reported thus far. First: early type, characterized by fetal edema and/or cystic hygroma and lethality in the second trimester; second: late type, characterized by absence of fetal edema and survival into the third trimester; third: Finnish type, characterized by hydrops fetalis from second trimester and beyond (de Die-Smulders et al., 1990a).

The pathogenesis of LMPS is closely related to AChR which comprises four different subunits that are controlled by their own genes. For example, the *CHRND* (MIM 100720) gene, which can cause four main types of congenital myasthenic syndrome (CMS): LMPS (MIM 253290), slow-channel CMS (MIM 616321), fast-channel CMS (MIM 616322), and AChR deficiency CMS (MIM 616323), is encoded by the *δ* subunit (Mishina et al., 1986; Engel et al., 2015). At the same time, to date, the genes *RAPSN* (MIM 601592), *nebulin* (*NEB*; MIM 161650), *CHRNA1* (MIM 100690), and *CHRNG* (MIM 100730) also cause varying pathogeneses in LMPS, with *CHRNG* mutations been observed in 30% of the cases (Morgan et al., 2006; Abdalla et al., 2017; Mohtisham et al., 2019). In adddition, previous studies have reported that LMPS can also be caused by uniparental disomy (UPD) of chromosome two carrying the pathogenic variant of *CHRND* (NM_000751.2) (Shen et al., 2018).

In view of the characteristic and non-specific nature of LMPS, this report aims to further study its etiology, clinical features, and the significance of prenatal examination in order to lay a solid foundation for early identification of LMPS in the future, which is helpful for prenatal diagnosis and counseling.

## Case report

A 34-year-old woman was admitted to the Prenatal Diagnosis Center of Guangzhou Women and Children’s Medical Center due to fetal nuchal cystic hygroma at 12 weeks of gestation. The pregnant woman denied alcohol, illicit drug, and tobacco usage. And that she had an adverse pregnancy in which the embryo had stopped developing without further detailed examination of the fetus. This fetus is the second child of the parents who are both healthy, safe and non-consanguineous. In addition, there was also no family history of hereditary disease.

### Prenatal ultrasound findings

**FIGURE 1:**
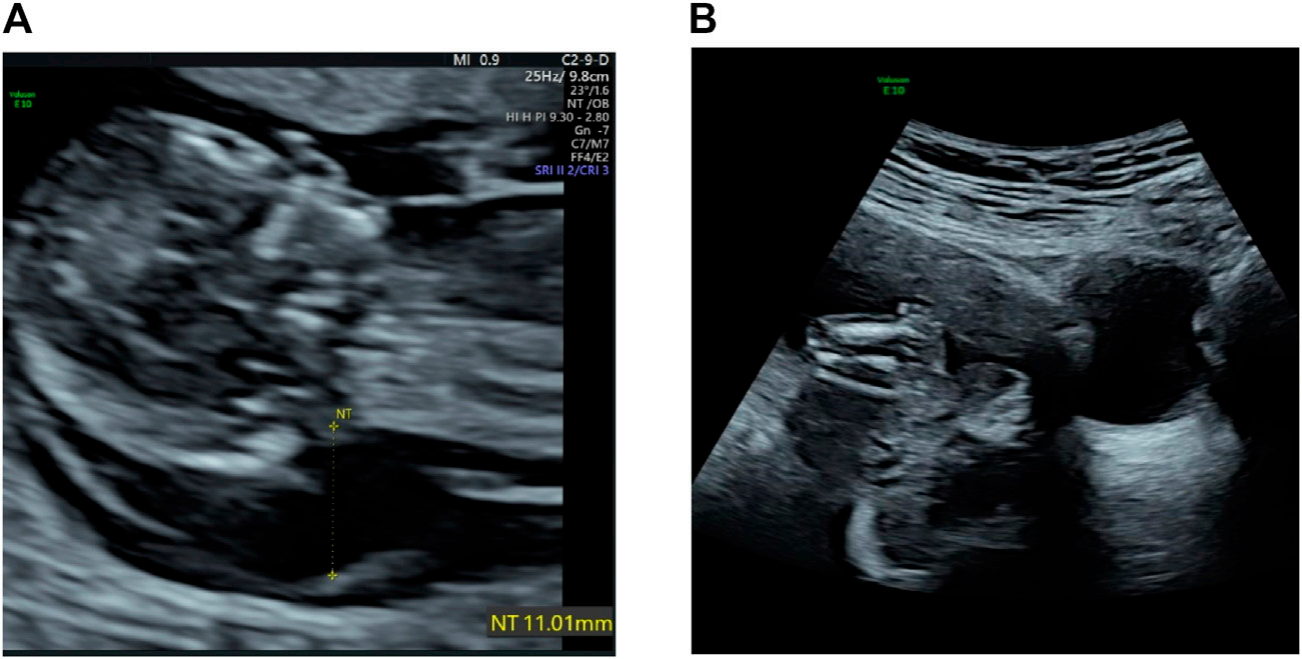
**(A)** Transabdominal ultrasound at 12 weeks shows a large cystic hygroma. **(B)** Flexion contracture of lower limb and edema.

**FIGURE 2:**
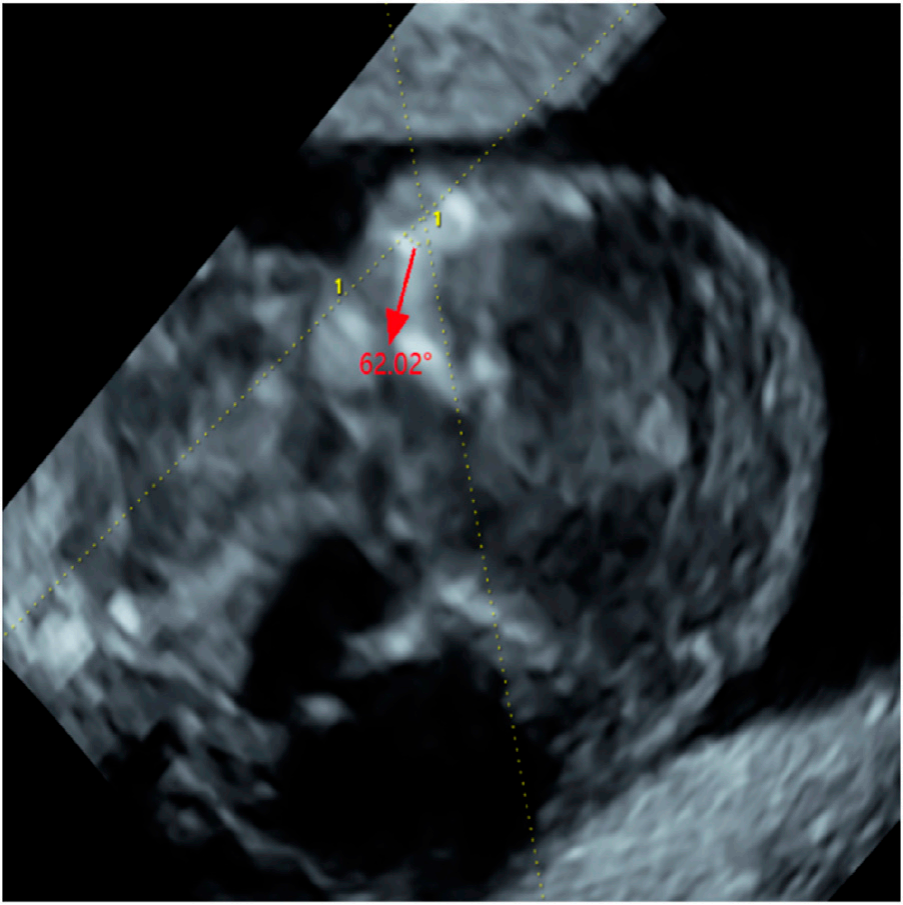
The measurement of Maxilla-nasion-mandible angle (MMF, 62.02°) (red arrow).

Abdominal two-dimensional (2D) ultrasound (US) revealed a single fetus at an estimated gestational age of 12 weeks 5 days on the basis of crown–rump length (CRL) of 64 mm. The fetus had a markedly increased nuchal translucency (NT) of 11 mm and absence of nasal bone (NB) (Figure 1A), edema and bilateral fixed flexion deformities of the knees and elbows (Figure 1B), vertical wrists, and a cyst in the right choroid plexus. Absence of limb motion was noted during the 20-min examination. In particular, micrognathia was observed, and we measured the mandibulomaxillary facial (MMF) angle as 62.02° by three-dimensional (3D) US and clearly observed contracture of upper limb (Figure 2). However, thickness of the placenta, cardiac structure, and middle cerebral artery peak systolic velocity (MCA-PSV) were normal.

### Phenotype of postpartum fetus

**FIGURE 3:**
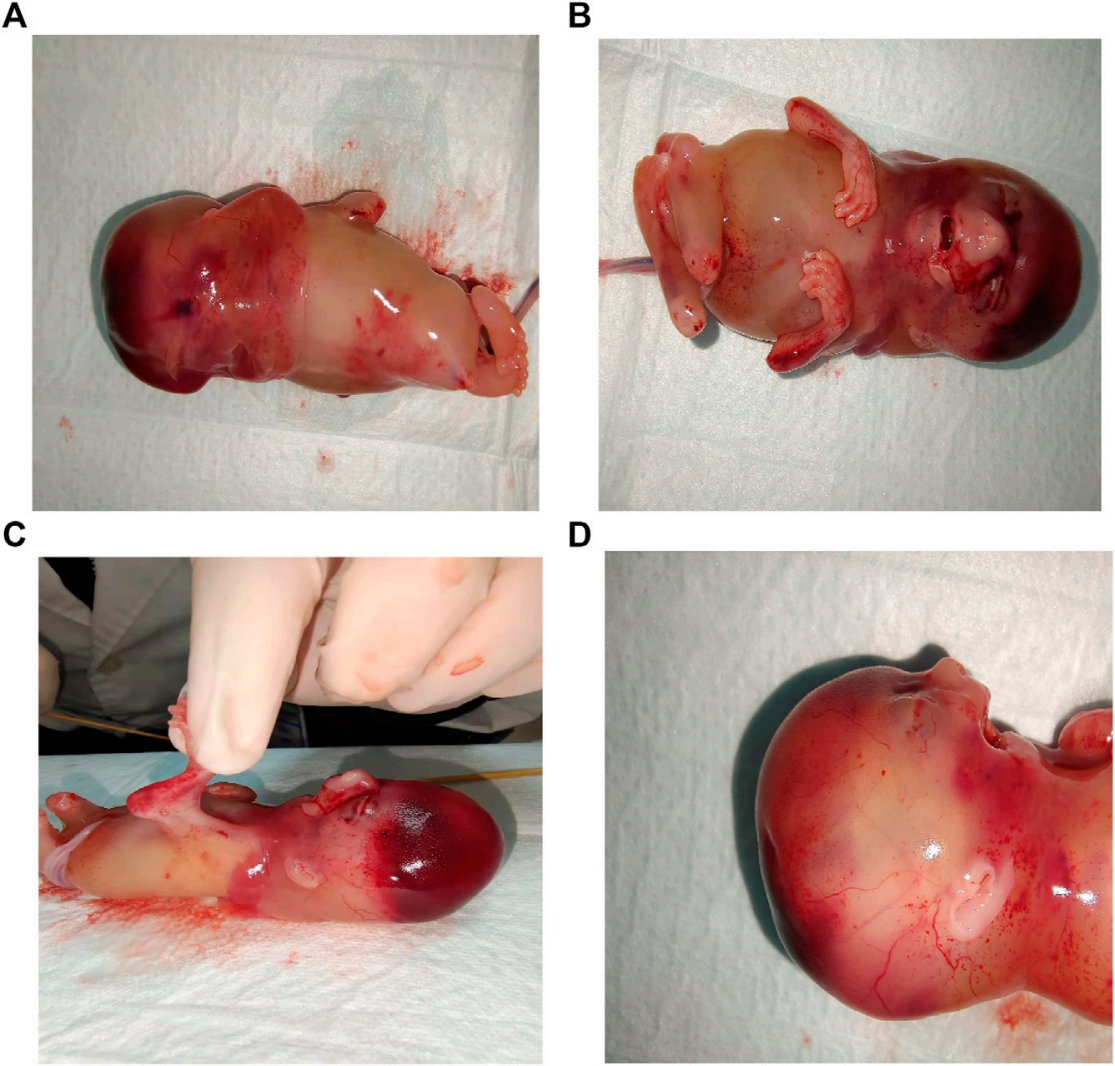
Stillbirth phenotypes: **(A)** Nuchal cystic hygroma. **(B)** Contractures of the extremities and vertical wrist. **(C)** Pterygium of antecubital and popliteal regions. **(D)** Side view: Pterygia of neck, low-set ears, micrognathia and gaping mouth.

As the prognostic impact of the condition is lethal, termination of the pregnancy with prostaglandin induction was proposed to the parents after consultation. Postartum fetus was noted to have multiple dysmorphic features (Figure 3), viz., nuchal cystic hygroma, contractures of the extremities, bilateral fetal talipes, subscalp hemorrhage, and short neck as well as spina bifida. Multiple pterygia were predominantly striking at the neck, antecubital, and popliteal regions. Facial abnormalities observed were micrognathia, low-set ears, cleft palate, and gaping mouth. Genitalia were that of a normal male.

### Whole-exome sequencing

Quantitative fluorescent-PCR (QF-PCR) and chromosomal microarray analysis (CMA) of the fetus were performed. And the couple were also advised to undergo whole-exome sequencing (WES) to further identify the etiology by prenatal consultation. Genomic DNA was randomly fragmented and purified using the magnetic particle method. WES was performed on an IIIumina HiSeq 2,500 sequencer (Illumina, San Diego, CA, United States) for a minimal of 10.14 Gb read-depth per case. Sequencing reads after quality control were aligned to the human reference genome by BWA (hg19). Nucleotide changes in the aligned reads were reviewed using NextGENe software (Version 2.4.1.2; SoftGenetics, State College, PA, United States). Novel variants were filtered against the 1,000 Genomes (http://www.1000genomes.org/), dbSNP (http://www.ncbi.nlm.nih.gov/snp), and Genome Aggregation databases (http://gnomad.broadinstitute.org/). Databases of ClinVar (version #372716), OMIM (version #602063.0005), ClinGen (version #CA5788214), as well as Human Gene Mutation were referred to in the analyses. Furthermore, the software Mutation Taster and PROVEAN were used to predict the impact of non-sense variant. For the in-frame deletion variants, the prediction tool was NNSplice. Common variants (population frequency <1% in gnomAD) were considered. Finally, PCR was performed to amplify the affected fragment of *CHRND* gene (NM_000751.2) using specific primers, and the purified PCR products were applied to Sanger sequencing to affirm the variant(s).

## Results

**FIGURE 4:**
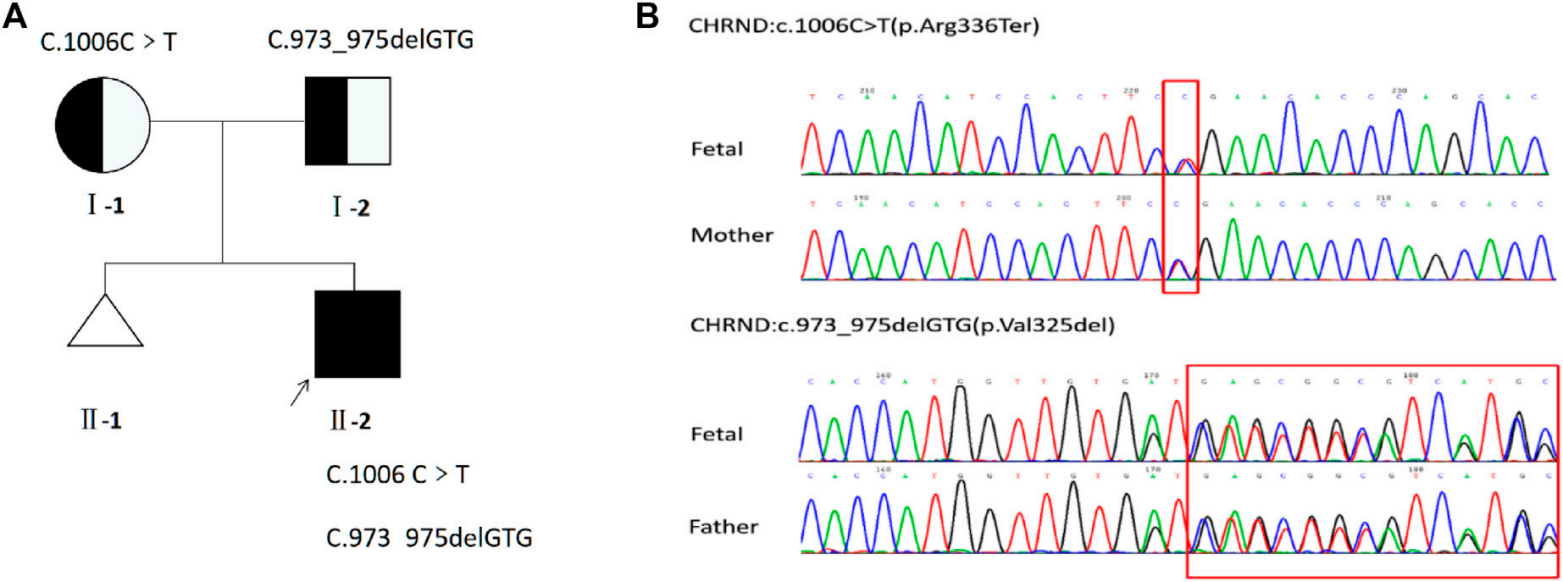
**(A)** Pedigree of the family. **(B)** Sequencing of *CHRND* gene (reference cDNA sequence, NM_000751.2) revealed two heterozygous variations, resulting in C to T substitution at nucleotide position 1006 [c.1006C>T p.(Arg336Ter)] and GTG deletion at nucleotide position 973 to 975 [c.973_975delGTG p.(Val325del)].

The results of QF-PCR and CMA were normal. And the minor allele frequency of c.1006C>T was 0.00002387 in gnomAD, which is also described in database dbSNP (rs754087173) and ClinVar (SCV002022550.1) but not recorded in the 1,000 Genomes databases. Moreover, the protein was truncated due to a non-sense variant, which caused a shift in the location of termination codon. And the in-frame deletion variant c.973_975delGTG resulted in protein length changes, which is not recorded in the gnomAD, dbSNP, and 1,000 Genomes databases. Based on the above mentioned outcomes and ACMG standards, one of the variants [NM_000751.2:c.1006C>T p.(Arg336Ter)] of *CHRND* gene, as maternal, was defined as likely pathogenic (LP:PVS1 + PM2) and the other [NM_000751.2:c.973_975delGTG p.(Val325del)], as paternal, was a variant of uncertain significance (VUS:PM2 + PM3 + PM4). Sanger sequencing of the fetus and parents further confirmed these results that parents were asymptomatic carriers of one variant (Figure 4).

## Previous cases

In the available literature, a total of four cases of LMPS caused by *CHRND* gene confirmed by US and genetic testing were reported from 2008 to 2018 (Table 1). Assuming that the information without mentioning sufficient in the articles were ruled out, it was revealed that only one case was found during early pregnancy which had four times of adverse pregnancy history, three of them may be LMPS by comprehensive analysis. And one showed micrognathia on the US but in the second trimester. Clinical details are summarized in Table 1.

**TABLE 1:** Summary of clinical manifestations in the current patients with *CHRND* gene variants.

Year	Michalk et al. (2008)			Shen et al. (2018)	Current case (2022)
Case	F1	F2		F3^f^	F4
Variant	NM_000751.2:c.234G>A(p.Trp57Ter)	NM_000751.2:c.1390C>T (p.Phe74Leu), NM_000751.2:c.283T>C (p.Arg443Ter)		NM_000751.2:c.236T>A (p. Ile79Lys), NM_000751.2:c.340G>C (p.Val114Leu)	NM_000751.2:c.973_975delGTG(p.Val325del), NM_000751.2:c.1006C>T(p.Arg336Ter)
Variant type	Homozygous non-sense	Compound non-sense-missense		Homozygous missense	Compound nonsense-in-frame deletion
Gender	M	M		M	M
Ethnicity	Turkish	German		NM	Chinese
Consanguinity	+	−		−	−
GA(week)	15^a^	13^a^	19^a^	31.5^b^	12^c^
Outcome	TOP	TOP	TOP	Death^d^	TOP
MA(yr)	NM	NM		NM	34
GPA	G3P1A1^e^	G5P0A4^e^	G6P0A5^e^	NM	G2P0A1^e^
Prenatal ultrasound findings					
Growth retardation	+	+	+	NM	−
Decreased movements	+	+	NM	NM	NM
Pterygia	NM	NM	NM	NM	−
Contracture	+	+	+	+	+
Polyhydramnios	NM	NM	NM	+	−
Edema/cystic hygroma	+/+	NM/+	+/+	NM	+/+
Absence of nassal bone	NM	NM	NM	NM	+
Cleft palate	NM	NM	NM	NM	-
Micrognathia	NM	NM	NM	NM	+
Low-set ears	NM	NM	NM	NM	−
Others features	NM	NM	NM	Absence of stomach bubble	Choroid plexus cyst
Phenotype of postpartum fetus					
Pterygia	+	+	+	+	+
Contracture	+	+	+	+	+
Edema/cystic hygroma	NM/NM	NM/NM	NM/NM	NM/NM	−/+
Cleft palate	NM	NM	NM	NM	+
Micrognathia	NM	+	+	+	+
Low-set ears	NM	+	+	NM	+
Scoliosis	+	NM	NM	NM	NM
Others features	Pectus excavatum;broad ribs, claviculae and os metatarsale; reduced muscle bulk/hypoplasia	NM	Facio cranial dysmorphism;down-slanting palpebral fissures; hypertelorism; a depressed nasal bridge	Small chest	Subscalp hemorrhage; short neck; bilateral fetal talipes; spina bifida

+, present; -, not present; GA, gestation age; TOP, termination of pregnancy; MA, maternal age; GPA, gravida para abortus; NM, not mentioned.

^a^ GW, of TOP.

^b^ GW, of amniocentesis.

^c^ GW, at US, diagnosis.

^d^ The fetus was delivered preterm and died shortly after birth.

^e^ adverse pregnancy history.

^f^ The variants originated from uniparental disomy (UPD) and the variants of c.340G>C (p.Val114Leu) was non-pathogenic.

## Discussion

LMPS is a rare disease and is fatal before birth or in the early neonatal period (HALL, 1984), and until the advent of prenatal diagnosis for causative genes, fetal abnormalities observed on US and autopsy had remained the only method to diagnose the disorder (Lockwood et al., 1988). As far as previous reports are concerned, nearly 22 LMPS cases (born or unborn) had increased during the decade before 2000 (Moerman et al., 1990; Meyer-Cohen et al., 1999). However, the data lacked reliability and comparability due to the absence of diagnostic results. Therefore, few cases of LMPS had been definitively diagnosed antenatally. WES gradually became a vital prenatal diagnostic technique for this kind of diseases (Todd et al., 2015), and the variants of the proband not reported before were then newly observed.

LMPS is caused by primary defects at any point in the motor system, mainly due to abnormalities in AChR, which are crucial for signaling between nerve cells and muscle cells (Hall, 1997; Morgan et al., 2006). During the fetal development, muscle-specific AChR is critical for signal transmission between nerve and muscle cells, and composes of four different subunits, two *α* subunits, one *β*, one *δ* and one γ/ε subunit, decided by *CHRNA1*(MIM 100690), *CHRNB1* (MIM 100710), *CHRND* (MIM 100720) and *CHRNG* (MIM 100730)/*CHRNE* (MIM 100725), respectively. And the *ε* subunit will replace the γ after around 33 weeks gestation (Mishina et al., 1986). The process of AChR formation, including assembly, clustering, anchoring, activation, and linking, must be rigorous to ensure that all subunits are functional during signaling. At the same time, this process requires the participation of many functional proteins, Such as musculoskeletal tyrosine kinase [MUSK (MIM 601296)], the muscle-intrinsic activator of MUSK named downstream of tyrosine kinase 7 [DOK7 (MIM 610285)], and the receptor-associated protein, *RAPSN* (MIM 601592) et al. (Sanes and Lichtman, 2001). In conclusion, abnormalities in any of the steps will cause the corresponding disease. For the report, the non-sense variant (c.1006C>T) causing a shift in the location of termination codon resulted in protein truncation, and the in-frame deletion variant (c.973_975delGTG) made the number of amino acids reduced, thus shortening the protein length. According to the InterPro database, both sites are Neurotransmitter-gatedion-channel transmembrane regions and conserved regions. The above results may affect *CHRND* gene, which in turn affects *δ* subunit, further affects AChR, and finally leads to LMPS. However, the above analysis needs to be further confirmed by more experiments.

In this case, LMPS caused by *CHRND* gene mutation in the fetus is a fatal disease and an AR disorder, which comes from the parents in the carrier state (Orpha, 2019). The pregnant woman had a history of missed abortion in the past, and although no further relevant diagnostic examinations were performed, it could not be ruled out that it was caused by this gene mutation. Eight phenotypes of the fetus reported in this report were consistent with the OMIM database (Omim, 2017), including the classic phenotypes of pterygium, nuchal cystic hygroma, edema, limb flexion and absence of limb motion, and others such as micrognathia, low-set ears and cleft palate. However, other unrecognized phenotypes may be limited by the small gestational week of the fetus, of the fetus, limited mobility, and the absence of further autopsy and/or imaging after labor induction. In general, the genotype and phenotype analysis are basically consistent, which is consistent with the diagnostic results. The mutation caused by this gene should be paid attention to and detailed examination should be performed.

A total of three families have previously been reported to have affected fetuses. In a Turkish family reported in 2008, the first pathogenic child did not receive further prenatal diagnosis, and the proband was the second child with LMPS, and the results showed that the *CHRND* gene suggested the first reported homozygous non-sense mutation. And in the German family, the genetic diagnosis of the fifth child was initiated, which indicated compound heterozygous mutation of *CHRND*, and the pregnancy was terminated in the first trimester, but the mother had already had two miscarriages and two pathogenic fetuses. The subsequent sixth abnormal fetus was identical to the fifth. The pregnant women chose to terminate the above pregnancies (Michalk et al., 2008). This family may not receive genetic counseling and diagnosis in time, which may result in multiple adverse pregnancy history, and cause great trauma to the pregnant woman and her family. Therefore, further counseling and examination should be performed after fetal abnormalities are found. Additionally, the genetic mutation of *CHRND* in the fourth case reported in 2018 occurred in UPD on chromosome 2 (Shen et al., 2018), which further complemented the pathogenesis of LMPS. The fifth case of *CHRND* gene (NM_000751.2) with novel variant causing LMPS was presented in this report, but there were some differences. LMPS can be diagnosed prenatally in the first trimester without pregnancy history of LMPS. Micrognathia and absence of NB were observed in the US at early pregnancy and cleft palate was noted in the postpartum fetus, which the other cases were not reported in detail. In addition, the typical phenotypes of the fetus, such as nuchal cystic hygroma and contracture of the limbs, were highly consistent between prenatal super and induced labor. Generalized analysis, the fetus was classified to the “early” form of LMPS, and new clinical features were observed that could aid in early identification and diagnosis. Howerer, the typical sign of pterygium was not found by prenatal US in all the above cases, which may be due to the fact that the limbs of the fetus are often flexed and the movement of the fetus is limited and the cystic hygroma in the neck. We need to further improve the level of US technology and knowledge of doctors, which is conducive to the early detection of fetal abnormalities.

This may be the first case of LMPS, thus far, in which the facial angle was measured by 3D US to identify micrognathia during early pregnancy. Maxilla–nasion–mandible angle (MNM), inferior facial angle (IFA), and frontal nasomental angle (FNM) were excluded due to the absence of NB, and MMF was selected. Compared with the critical value of previous statistical results in the first trimester, the value was less than normal [MMF 103.1°–114.5° (Borenstein et al., 2007)], which provides an objective reference index for micrognathia. In addition, the various modes of 3D US compensate for the defects in 2D US so that the fetal structure is clear and easy to observe (Platt et al., 2006). This facilitates the early detection of more abnormal phenotypes in the fetus to attain a definitive diagnosis and determine the next management measures.

It has been demonstrated that the autopsy results are vital for further analysis of the genetics and phenotype of prenatal VUS and LP. Based on the comprehensive analyses of prenatal and postnatal phenotypes, one of the mutations (c.1006C>T) was defined as pathogenic (P: PVS1 + PM2 + PP4) and the other (c.973_975del) was LP (PM2 + PM3 + PM4 + PP4), which reversely confirmed the prenatal phenotype and disease outcome corresponding to *CHRND* gene (NM_000751.2). In addition, the parents are made aware that the risk of the offspring developing the disease is 25% after prenatal consultation. They should also be offered preventive measures. As the primary prevention strategy, the couple should consider undergoing adjuvant reproductive technology (ART). As the secondary prevention strategy, routine examination (consultation, ultrasound, and molecular diagnosis) would be indispensable for pregnant women with abnormal fetuses, and then timely intervention to choose the best course of treatment.

In conclusion, we have reported compound heterozygous variants apart from the variation spectrum of the *CHRND* (NM_000751.2) gene and a new diagnosis method for the early recognition of micrognathia, which is vital to provide appropriate genetic counseling to the parents. And micrognathia and nuchal cystic hygroma can be identified in the first trimester, which should be focused on, and clinical intervention should be performed as soon as possible.

## Data Availability

The datasets for this article are not publicly available due to concerns regarding participant/patient anonymity. Requests to access the datasets should be directed to the corresponding author.
